# Probing the Occurrence of Soluble Oligomers through Amyloid Aggregation Scaling Laws

**DOI:** 10.3390/biom8040108

**Published:** 2018-10-04

**Authors:** Alexandra Silva, Zsuzsa Sárkány, Joana S. Fraga, Pablo Taboada, Sandra Macedo-Ribeiro, Pedro M. Martins

**Affiliations:** 1 IBMC—Instituto de Biologia Molecular e Celular, Universidade do Porto, 4200-135 Porto, Portugal; a.silva@ibmc.up.pt (A.S.); zsarkany@ibmc.up.pt (Z.S.); Joana.Fraga@ibmc.up.pt (J.S.F.); sribeiro@ibmc.up.pt (S.M.-R.); 2 Instituto de Investigação e Inovação em Saúde, Universidade do Porto, 4200-135 Porto, Portugal; 3 ICBAS—Instituto de Ciências Biomédicas Abel Salazar, Universidade do Porto, 4050-313 Porto, Portugal; 4 Área de Física de la Materia Condensada, Facultad de Física, Universidad de Santiago de Compostela, 15782 Santiago de Compostela, Spain; pablo.taboada@usc.es; 5 Instituto de Investigación Sanitaria (IDIS), 15706 de Santiago de Compostela, Spain

**Keywords:** protein aggregation, amyloid, soluble oligomers, kinetic analysis, nucleation

## Abstract

Drug discovery frequently relies on the kinetic analysis of physicochemical reactions that are at the origin of the disease state. Amyloid fibril formation has been extensively investigated in relation to prevalent and rare neurodegenerative diseases, but thus far no therapeutic solution has directly arisen from this knowledge. Other aggregation pathways producing smaller, hard-to-detect soluble oligomers are increasingly appointed as the main reason for cell toxicity and cell-to-cell transmissibility. Here we show that amyloid fibrillation kinetics can be used to unveil the protein oligomerization state. This is illustrated for human insulin and ataxin-3, two model proteins for which the amyloidogenic and oligomeric pathways are well characterized. Aggregation curves measured by the standard thioflavin-T (ThT) fluorescence assay are shown to reflect the relative composition of protein monomers and soluble oligomers measured by nuclear magnetic resonance (NMR) for human insulin, and by dynamic light scattering (DLS) for ataxin-3. Unconventional scaling laws of kinetic measurables were explained using a single set of model parameters consisting of two rate constants, and in the case of ataxin-3, an additional order-of-reaction. The same fitted parameters were used in a discretized population balance that adequately describes time-course measurements of fibril size distributions. Our results provide the opportunity to study oligomeric targets using simple, high-throughput compatible, biophysical assays.

## 1. Introduction

The deposition of amyloid fibrils in the brain is a pathological hallmark of several different neurodegenerative disorders, yet the pathogenic role of these insoluble aggregates is not fully understood [1]. On the other hand, there is now substantial in vivo evidence of amyloidogenic proteins also forming small soluble oligomers that spread to neighboring cells and induce downstream processes associated with neurodegeneration [1,2]. Chemical kinetics, a classical cornerstone for drug discovery [3], is hardly applicable to the study of this new and pre-eminent target [4,5,6], in part due to the lack of straightforward methods to monitor the formation of a highly heterogeneous group of species ranging from protein dimers to complex *n*-mers [7,8]. In contrast, extensive research has been devoted to protein aggregation kinetics based on the characteristic tinctorial properties of amyloid fibrils [9].

An important step towards the kinetic quantification of off-pathway aggregation was taken after the observation of protein precipitation occurring in parallel with the formation of amyloid fibrils of lysozyme [10]. Before, kinetic analysis of amyloid aggregation of the islet amyloid polypeptide (IAPP) suggested the formation of intermediate on- and off-pathway phases during IAPP fibrillogenesis [11]. The presence of non-amyloidogenic species produces perceptible deviations from the time evolution of the amyloid signal expected for the generic nucleation and growth processes of the phase transition:

(1) $$\;\alpha =1-\frac{1}{k_{b}\left[\exp\left(k_{a}t\right)-1\right]+1}\;$$

where α is the normalized amyloid conversion, and $k_{a}$ and $k_{b}$ are combinations of elementary rate constants [12]. One of the kinetic signatures found to be associated with off-pathway aggregation was the unusually weak dependence of the lag phase duration on the initial concentration of lysozyme [10]. Similar behaviors observed with other protein models have provided the basis for varied interpretations of the amyloid aggregation mechanism encompassing, for example, Michaelis-Menten-like saturation of the elongation step [13], complex sub-steps of nucleation and growth [14], stochastic fluctuations in the nucleation time [15], and the suppression of fibril fragmentation at high fibril concentrations [16]. Unlike these possible explanations for the underperforming scaling laws, off-pathway aggregation can be directly investigated by analytical and microscopic techniques such as those used to identify insoluble aggregates of lysozyme [10], and later on, soluble oligomers of ataxin-3 [17], and metastable oligomers of Aβ40 and Aβ42 peptides [18,19].

Because the formation of soluble and insoluble assemblies is fed by a common pool of protein monomers, we propose that amyloid fibrillation kinetics can be used to reveal the presence of the parallel oligomeric pathway. To test this hypothesis, we chose two systems, human insulin and ataxin-3, for which the fibrillation kinetics have been measured under conditions of known oligomeric composition. Insulin is a protein hormone existing in solution in a thermodynamic equilibrium of monomers, dimers, tetramers, hexamers and higher-order oligomers [20,21]. Changes in the protein molecular structure induced by low pH, high temperature or the presence of organic solvents lead to the formation of amyloid fibrils through the direct association of insulin monomers [20], or by the assembly of intermediate on-pathway oligomers [22]. Ataxin-3 is a multi-domain protein with a globular Josephin domain and a C-terminal flexible tail containing a polyglutamine (polyQ) repeat whose expansion ultimately causes Machado–Joseph disease. Ataxin-3 aggregation involves an initial step mediated by the Josephin domain, and a second step dependent on the expanded polyQ tract that accelerates protein aggregation and promotes the formation of mature amyloid fibers [23,24]. The analysis of the thioflavin-T (ThT) binding assay run at different concentrations of human insulin and ataxin-3 uncovers mechanistic aspects of the oligomeric and fibrillar pathways. The distinct aggregation mechanisms predicted for each protein are experimentally validated by time-course dynamic light scattering (DLS) measurements.

## 2. Materials and Methods

### 2.1. Protein Preparation

Human insulin purchased from Sigma-Aldrich (Saint Louis, MO, USA) (I2643) was dissolved without further purification in 20% acetic acid 0.5 M NaCl (pH 1.8) to a final concentration of 5 mg/mL. Before incubation, samples were filtered with 0.22 μm syringe filter units (Millex-GV, Millipore, Cork, Ireland). Non-expanded ataxin-3 was expressed and purified as previously described [17].

### 2.2. Transmission Electron Microscopy 

Transmission electron microscopy (TEM) visualization of insulin and ataxin-3 fibrils was performed using a TEM JEM-1400 (JEOL, Tokyo, Japan) at an accelerating voltage of 80 kV. 100 μL samples of 5 mg/mL insulin were incubated for 6 h at 45 °C without mechanical shaking in 1.5 mL eppendorf tubes (DNA LoBind, Eppendorf AG, Hamburg, Germany). 700 μL samples of 5 μM (0.218 mg/mL) ataxin-3 in 20 mM sodium phosphate pH 7.5, 150 mM NaCl, 1 mM dithiothreitol (DTT) were incubated in 1.5 mL eppendorf tubes (DNA LoBind, Eppendorf AG, Hamburg, Germany) for 65 h at 37 °C without mechanical shaking. Protein samples were diluted in water (1:20 for insulin and 1:10 for ataxin-3), adsorbed to carbon-coated 200 mesh nickel grids (FCF300-NI, Electron Microscopy Sciences, Hatfield, PA, USA), negatively stained with 2% (w/v) uranyl acetate, dried and observed at a magnification of 80,000–100,000×.

### 2.3. Dynamic Light Scattering 

Dynamic light scattering measurements were performed using an ALV/DLS/ SLS-5000F, SP-86 goniometer system (ALV-GmbH, Langen, Germany) equipped with a CW diode-pumped Nd:YAG solid-state Compass-DPSS laser with a symmetrizer (Coherent Inc., Santa Clara, CA, USA). The laser operates at 488 nm with an output power of 400 mW. The intensity scale was calibrated against scattering from toluene. 700 μL samples of 5 mg/mL insulin were incubated in glass cuvettes at 45 °C without mechanical shaking and periodically analyzed at a scattering angle 90° to the incident beam. Hydrodynamic radii of the particles in solution were estimated from the diffusion coefficient(s) delivered from CONTIN analysis [25]. Discontinuous auto-correlation functions were not considered for CONTIN analysis.

## 3. Results and Discussion

**Figure 1:**
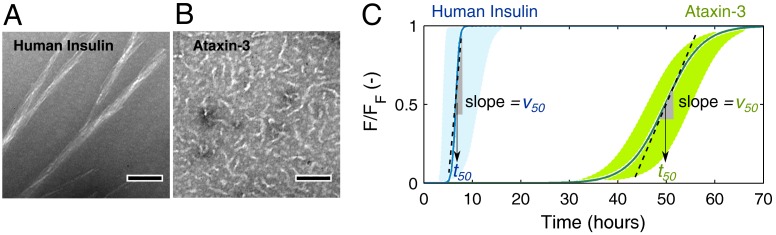
Case study examples of human insulin and ataxin-3 aggregation. Transmission electron microscopy (TEM) micrographs of negatively stained fibrils of (**A**) 5 mg/mL human insulin and (**B**) 5 μM (0.218 mg/mL) ataxin-3 captured after 6 h and 65 h incubation, respectively (scale bars, 100 nm). (**C**) Schematic amyloid fibrillation curves representing the progress of normalized thioflavin-T (ThT) fluorescence ($F/F_{F}$ ) during the aggregation of human insulin and ataxin-3 in the range of protein concentrations studied by Foderà et al. [26] and Silva et al. [17], respectively. The half-life coordinates $t_{50}$ and $v_{50}$ are indicated by the arrows and by the slopes of dashed lines, respectively.

For the experimental conditions adopted in each model protein, human insulin and ataxin-3 produce fibrillar species with distinct morphologies (Figure 1A,B) and at markedly different aggregation rates (Figure 1C). Long, straight filaments of human insulin are formed much faster than the small, worm-like fibrils of ataxin-3, thereby suggesting that phase transition mechanisms are differently affected by the fibril elongation step. Chemical kinetic analysis pinpoints these differences, and reveals how the presence of soluble oligomers influences each type of protein aggregation curves. The quantitative methods proposed here are expected to contribute to the identification of mechanistic changes provoked, e.g., by the presence of aggregation modulators or by different conditions of temperature, pH, ionic strength, etc.

### 3.1. Mechanistic Analysis of Insulin Aggregation

**Figure 2:**
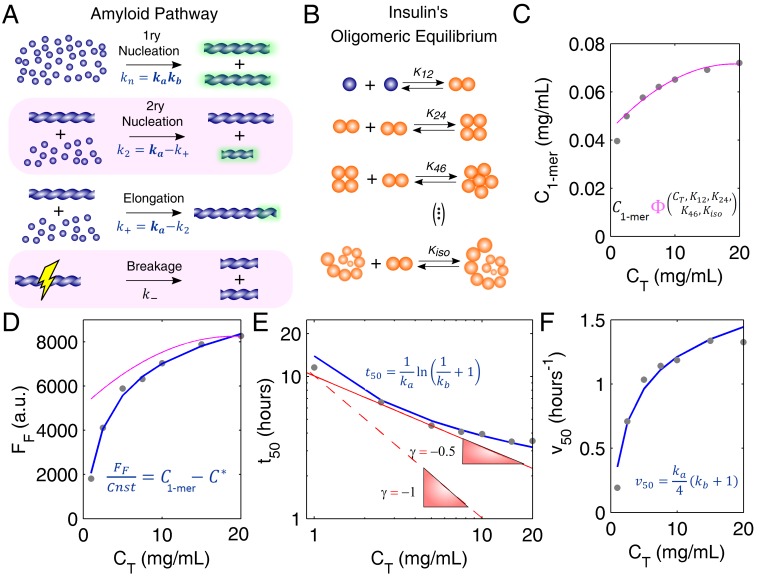
Aggregation pathways of human insulin investigated through amyloid fibrillation kinetics. (**A**) Reaction steps and corresponding rate constants participating in the amyloid pathway. Green glows represent an increase in the mass of fibrils. This variation is detected by amyloid binding assays and can be used to estimate two parameters, $k_{a}$ and $k_{b}$, consisting of combinations of the other rate constants (see text for details). (**B**) Oligomeric equilibrium of insulin as determined by Bocian et al. [21] using 2D and pulsed field gradient spin echo (PFGSE) nuclear magnetic resonance (NMR) ($K_{12}=4.9\times 10^{5}$, $K_{24}=5.0\times 10^{4}$, $K_{46}=2.7\times 10^{3}$ and $K_{\mathrm{iso}}=1.35\times 10^{4}$ ). (**C**) Concentration of insulin monomers ($C_{1-\mathrm{mer}}$ ) predicted by the oligomeric equilibrium (**B**) for the values of total protein concentration ($C_{T}$ ) used in (**D**–**F**) (symbols). Pink line: polynomial fit to the data. (**D**–**F**) Reaction scaling laws measured by Foderà et al. [26] (symbols) and predicted by the model equations shown in blue for the monomer concentrations estimated in (**C**) (solid blue lines). (**D**) The final ThT fluorescence ($F_{F}$) is a direct proportion of supersaturation $\Delta C=C_{1-\mathrm{mer}}-C^{*}$ (proportionality constant $cnst=1.95\times 10^{5}$ ) for an inferred solubility value of $C^{*}=0.029$ mg/mL. Pink line: The polynomial fit in (**C**) is used to estimate $F_{F}\;$ without the solubility correction ($cnst=1.15\times 10^{5}$ and $C^{*}=0)$. (**E**) Double-logarithmic plot of half-life coordinate $t_{50}$ as a function of $C_{T}$. Red lines: Limit scaling exponents |γ| of 1 (dashed line) and 0.5 (solid line) are still too high to represent the measured trend. (**E**,**F**) Both $k_{a}$ and $k_{b}$ are considered first-order dependent on ΔC (fitted values: $k_{a}=1.34\times 10^{2}\Delta C$ h^−1^ and $k_{b}=2.41\times 10^{-7}\Delta C$ ). Measured data were adapted with permission from Foderà et al. [26]. Copyright 2017 American Chemical Society.

In the simplified mechanism represented in Figure 2A, the elementary intermediate steps participating in the primary nucleation, secondary nucleation and elongation of insulin fibrils are summed up into the overall rate constants $k_{n}$, $k_{2}$, and $k_{+}$, respectively [17]. The sigmoidal (rather than hyperbolic) progress curve of insulin fibrillation (Figure 1C) points to low values of the parameter $k_{b}=k_{n}/k_{a}$, which gives the relative weight of primary nucleation over the autocatalytic steps of secondary nucleation and elongation ($k_{a}=k_{2}+k_{+}$) [10]. The fast elongation rates suggested by the morphology of insulin fibrils (Figure 1A) are confirmed by the high value of $k_{a}$ associated to the steep burst phase (and high $v_{50}$ value) in Figure 1C. The specific weight of secondary nucleation and elongation in determining the value of $k_{a}$ cannot be distinguished from single progress curve analysis because these steps follow similar rate laws [17]. Moreover, since fibril breakage (rate constant $k_{-}$) does not change the total mass of ThT-positive filaments but only their number [17], complementary measurements of fibril size distributions are required to directly assess the role of the breakage step.

Prior knowledge of the protein oligomerization state is required before we can move into the deeper levels of the different aggregation pathways [27]. The oligomerization equilibrium of human insulin (Figure 2B) has been characterized by Bocian et al. [21] using 2D and pulsed field gradient spin echo (PFGSE) nuclear magnetic resonance (NMR). It is, therefore, possible to estimate the availability of insulin monomers under conditions of total protein concentration, presence of zinc, and acidic pH that are similar to those adopted by Foderà et al. [26] while measuring amyloid fibrillation kinetics. Based on the knowledge of the values of the monomer concentration $C_{1-\mathrm{mer}}$ (Figure 2C), peculiar scaling laws of equilibrium (Figure 2D) and kinetic (Figure 2E,F) parameters can be explained using a number of fitted parameters commensurate with the number of independent observations. As an indicator of the amount of amyloid fibrils produced, the final ThT fluorescence intensity ($F_{F}$) (pink line in Figure 2D) is not directly determined by the total protein available (closed circles in Figure 2D) or even by the monomer concentration alone. Since protein aggregation takes place until the monomer concentration $C_{1-\mathrm{mer}}$ equals the thermodynamic solubility $C^{*}$, the value of $F_{F}$ reflects the difference $\left(C_{1-\mathrm{mer}}-C^{*}\;\right)$ otherwise known as supersaturation (ΔC) [12]. This is illustrated in Figure 2D (blue line) with no other fitting parameters than the fluorescence proportionality constant (in arbitrary units) and the insulin solubility, which is a measurable quantity. Besides confirming amyloid fibrillation as a phase transition process driven by supersaturation, the $F_{F}$ scaling law is consistent with a mechanism of monomer addition admitting no supplementary contribution from pre-existing soluble oligomers to the final ThT fluorescence signal. Consequently, the amyloid pathway (Figure 2A) and the oligomeric equilibrium (Figure 2B) are found to take place over distinct timescales, with insulin monomers being consumed by the first process at much faster rates than they are produced by the second.

The separation of timescales simplifies the application of analytic model equations that were originally derived by assuming the soluble protein fully dissociated [12]. Theoretical curves of $t_{50}$ and $v_{50}$ vs. protein concentration can be computed using the equations in Figure 2E,F (see Appendix A for details), after expressing $k_{a}$ and $k_{b}$ as a function of ΔC (and of $C_{1-\mathrm{mer}}$). If, as it seems to be the case of insulin, fibril elongation predominates over secondary nucleation (i.e., $k_{a}\approx k_{+}$ and $k_{b}\approx k_{n}/k_{+}$), then both $k_{a}$ and $k_{b}$ are proportional to the initial supersaturation (∝ΔC) considering that [10,12]:

(2) $$\;\{\begin{array}{c} k_{+}\propto \Delta C\\ k_{n}\propto \Delta C^{2} \end{array}\;$$

These simple premises and two model parameters are sufficient to elucidate the unconventionally weak $C_{T}$-dependence of $t_{50}$ (Figure 2E) as being the result of the lower molar fractions of insulin monomer observed for higher protein concentrations (Figure 2C). If the associated states of soluble insulin were ignored, the lower limits usually admitted for the absolute scaling factor |γ| would be too high to reproduce the measured trend in Figure 2E (red lines). Remarkably, the set of parameters, $k_{a}$ and $k_{b}$ fitted to the lag-time scaling data in Figure 2E are the same as those that describe the aggregation rate data in Figure 2F (Appendix B). In both cases, the used value of $C^{*}$ is the one resulting from the interpretation of Figure 2C. Far from being redundant, the confirmation of kinetic predictions by different and independent measurements provides unequivocal evidence that the present theoretical framework, with only two model parameters, is indeed valid.

### 3.2. Mechanistic Analysis of Ataxin-3 Aggregation

**Figure 3:**
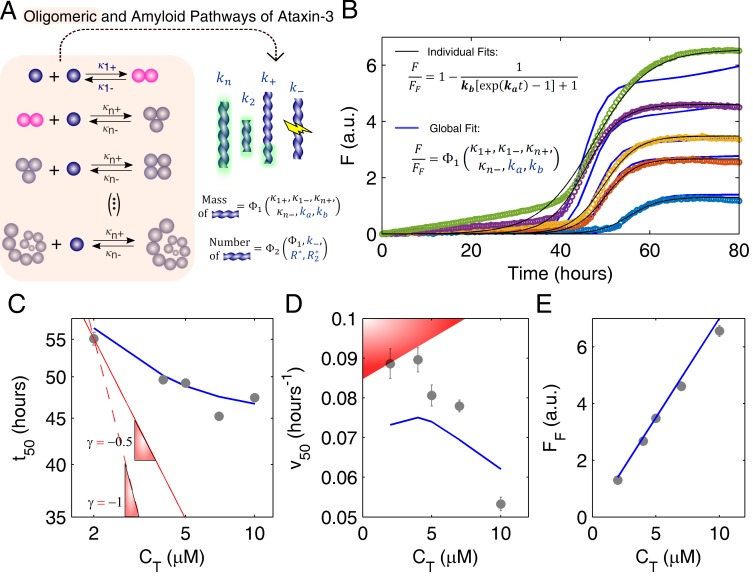
Aggregation pathways of ataxin-3 investigated through amyloid fibrillation kinetics. (**A**) The oligomeric and amyloid pathways take place simultaneously. The rate constants of oligomer formation/dissociation were previously determined ($\kappa_{1+}=7.99\times 10^{-4}$ μM^−1^ h^−1^, $\kappa_{1-}=9.73$ h^−1^, $\kappa_{n+}=0.167$ μM^−1^ h^−1^, and $\kappa_{n-}=0.775$ h^−1^) [17]. The steps of amyloid fibril formation are the same as in Figure 2A. The mass of amyloid fibrils is a function of only $k_{a}$ and $k_{b}$, whereas the number of filaments is also influenced by fibril breakage and by the critical size of fibrils formed by primary and secondary nucleation ($R^{*}$ and $R_{2}^{*}$, respectively). (**B**) Symbols: ThT fluorescence increase measured for ataxin-3 concentrations of (from top to bottom) $C_{T}=10$ μM, 7 μM, 5 μM, 4 μM and 2 μM [17]. Lines: individual (black) and global (blue) fittings of the experimental data by Equations (1) and (S7), respectively. Fitting statistics given in Figure S1A. Global fitting: $k_{a}=0.364C_{T}^{n_{2}}$ h^−1^, $k_{b}=2.91\times 10^{-10}C_{T}^{2-n_{2}}$ and $n_{2}=0.160$ ). (**C**–**E**) Reaction scaling laws corresponding to the kinetic measurements (symbols) and global fitting (blue lines) shown in (**B**). (**C**) Double-logarithmic plot. Red lines: Limit scaling exponents |γ| of 1 (dashed line) and 0.5 (solid line) are still too high to represent the measured trend. (**D**) Red-shadowed area: typically, $v_{50}$ is positively correlated with $C_{T}$ (and with $t_{50}^{-1}$ ) [29]. Measured data were adapted with permission from Silva et al. [17]. Copyright 2018 John Wiley and Sons.

The study of ataxin-3 aggregation follows the same underlying principle that was adopted for human insulin, and has a similar purpose: to show how traditional kinetics can be markedly distorted by the presence of soluble oligomers. As in the case of other polyQ-repeat proteins [28], the formation of ataxin-3 fibrils and the dissociation of ataxin-3 oligomers occur simultaneously (Figure 3A), and thus, timescale separation cannot be assumed as a simplifying hypothesis. Supported by DLS, size-exclusion chromatography and TEM data, a detailed account of the different steps shown in Figure 3A was recently provided [17], including quantitative estimations of the rate constants $\kappa_{1+}$, $\kappa_{1-}$, $\kappa_{n+}$, $\kappa_{n-}$ characterizing the elementary steps of ataxin-3 oligomerization. The worm-like fibrils shown in Figure 1B are predominantly formed by secondary nucleation ($k_{a}\approx k_{2}$) and primary nucleation $\left(k_{b}\approx k_{n}/k_{2}\right)$, with minor contributions from the fibril elongation ($k_{+}\approx 0$) and fibril breakage ($k_{-}\approx 0$) steps [17]. The measured effect of protein concentration on the ThT fluorescence progress curves (Figure 3B, symbols) is not fully assessed if the oligomeric pathway is not taken into account; on the whole, the black lines in Figure 3B are indicative of good numerical fits, yet they are based on Equation (1), which ignores the occurrence of the parallel reactions of soluble oligomer formation/dissociation. Regardless of how elaborated the theoretical model can be, the fitted parameters are, in this limited scenario, comparable to semi-empirical coefficients showing no evident fundamental meaning. In the illustrative case of Figure 3B, the empirically determined values of $k_{a}$ and $k_{b}$ would follow a proportional relationship with protein concentration, which is not reconcilable with established theories (Figure S1).

Instead of using the amyloid fibrillation model in its closed form solution, the original differential equation, was solved simultaneously with the oligomerization rate equilibrium, Equations (A6) and (A7) (Appendix A), and then fitted to the ThT fluorescence progress curves (Figure 3B, blue lines). Although computationally more demanding than the approach followed with human insulin, the number of degrees of freedom remains unusually low as regards to complex biophysical problems: three independent scaling laws of $t_{50}$ (Figure 3C), $v_{50}$ (Figure 3D) and $F_{F}$ (Figure 3E) are used to estimate no other unknowns but the scaling constants associated to $k_{a}$ and $k_{b}$. Unlike the case of insulin, the value of ataxin-3 solubility is known beforehand to be very low ($C^{*}\approx 0$) as evidenced by values of monomer concentration lower than the detection limits under equilibrium conditions [17]. In contrast, since the autocatalytic rate constant of ataxin-3 is determined by the secondary nucleation step ($k_{a}\approx k_{2}$), a scaling exponent $n_{2}$ is now introduced to account for the poorly understood $k_{2}$ vs. ΔC relationship:

(3) $$\;\{\begin{array}{c} k_{2}\propto \Delta C^{n_{2}}\\ k_{n}\propto \Delta C^{2} \end{array}\;$$

In practice, different fitted parameters are provided by the individualized analysis of each progress curve in Figure 3B (black lines), whereas the global fit (blue lines) requires a single set of rate constants $k_{a}$ and $k_{b}$ to model both the aggregation assay and its scaling laws (Figure 3C–E). The better goodness-of-fit statistics of the former procedure (Figure S1) is not surprising since, as in the case of overparameterized problems, the individual numerical analysis is not cross-validated and tends to overfit the experimental error, therefore, compromising the model’s predictive power [17,30,31,32]. The global fitting confirms that pre-determined oligomerization constants can be integrated in aggregation reaction networks to explain highly peculiar kinetics, such as the very weak $C_{T}$-dependence of $t_{50}$ (Figure 3C), and notably, the negative $C_{T}$-dependence of $v_{50}$ (Figure 3D). Although a more conventional result in the absence of quenching phenomena [33,34], the linear scaling law of the end-point ThT fluorescence (Figure 3E) is explained by the dissociation of ataxin-3 oligomers occurring in the same time scale as amyloid fibrillation. The observed straight line crossing the origin also indicates that the soluble protein was converted into amyloid-like fibrils without the occurrence of significant monomer degradation during incubation [17]. The complex, yet self-consistent behaviors of half-life and end-point readings cross-validate the molecular-level implications arising from the definition of the secondary nucleation rate constant ($k_{2}$), and particularly, from the obtained value of the scaling exponent $n_{2}$ close to 0. A direct comparison with the fibril elongation step would suggest a first-order dependence of $k_{2}$ on the initial supersaturation ΔC since both rates linearly increase with the instantaneous values of supersaturation and fibril mass [10,17]. However, more than just a collisional rate coefficient, $k_{2}$ is an overall rate constant accounting for the rate-limiting steps leading to the formation of secondary nuclei [35]. According to classical nucleation theory [36], the nucleation promoting effect elicited by higher supersaturation levels (and lower energetic barriers for phase transition) can be, in part, counteracted by the concomitant decrease in the critical sizes of the primary ($n_{1}^{*}$) and secondary ($n_{2}^{*}$) nucleus. This extra contribution, which is not evident for primary nucleation of amyloid fibrils [12], seems relevant for the secondary nucleation of ataxin-3. Somewhat undervalued in regard to induction time measurements, half-life aggregation rates $v_{50}$ (or, equivalently, maximum aggregation rates) offer the opportunity to identify the predominant autocatalytic process. Whilst the scaling of $t_{50}$ is greatly influenced by primary nucleation, the scaling of $v_{50}$ is determined by the balance between elongation and secondary nucleation rates, with the effect of $C_{T}$ getting weaker as secondary nucleation becomes more important. Therefore, and similarly to what was concluded for insulin, the measured scaling laws of ataxin-3 aggregation are determined by the fibrillation mechanism itself and by the presence of thermodynamically stable, soluble aggregates that further deplete the concentration of free monomer in solution.

### 3.3. Model Predictions Are Further Confirmed by Size Distribution Analysis of Insulin Aggregation

**Figure 4:**
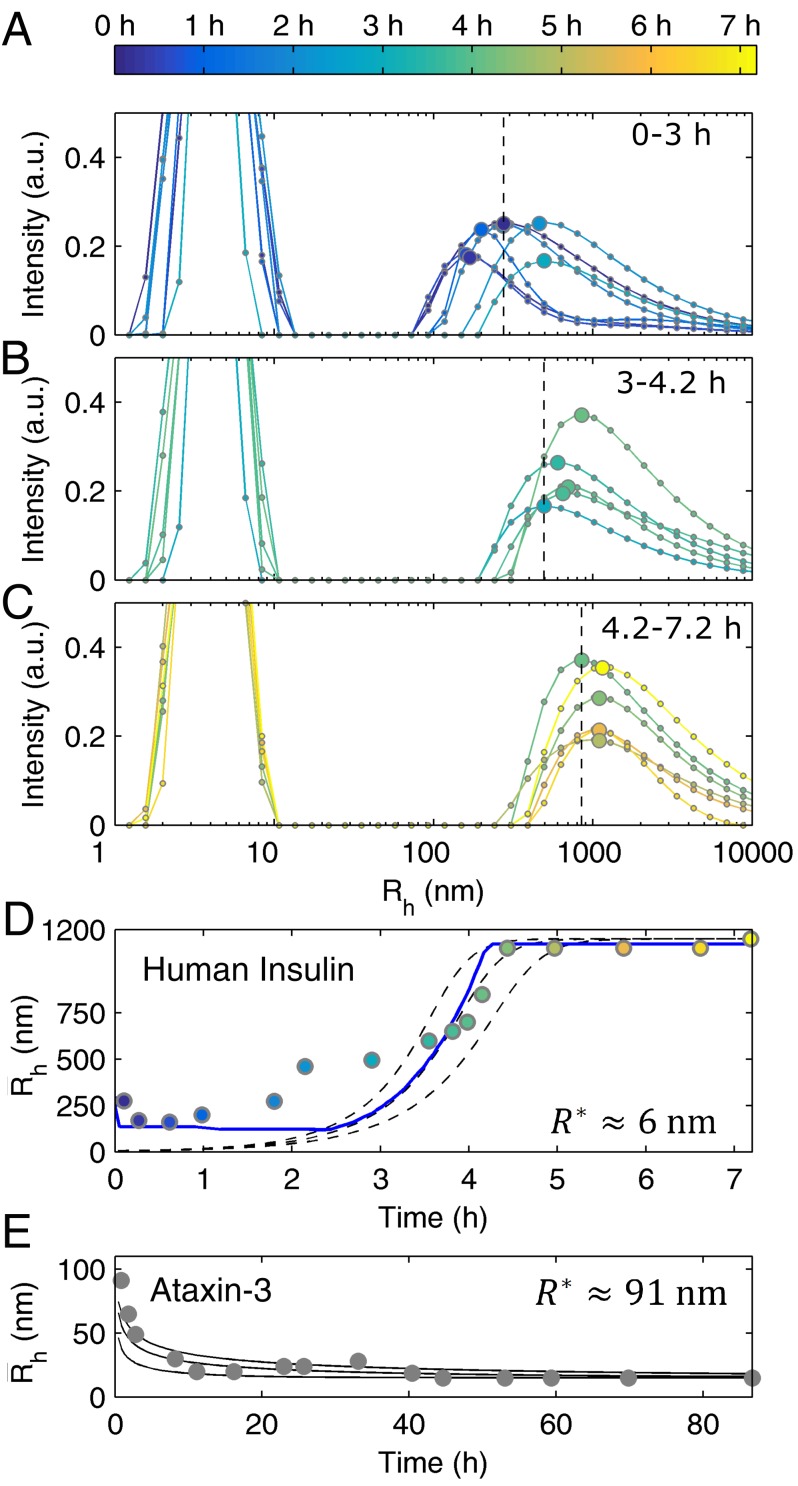
Time-course DLS analysis of human insulin aggregation—differences and common aspects with ataxin-3. (**A**–**C**) Symbols connected by lines: intensity-based size distributions measured at different time points as indicated by the color bar in (**A**). Larger symbols: values of the hydrodynamic radius ($R_{h}$) used as estimates of the mean size (${\bar{R}}_{h}$ ) of insulin fibrils. Vertical dashed lines: visual reference of the first ${\bar{R}}_{h}$ value of each panel. (**D**) Measured (symbols) and simulated (lines) time evolution of ${\bar{R}}_{h}$. Dashed lines: representations of Equation (4) using values of $k_{a}=4.13$ h^−1^ and $k_{b}=6.90\times 10^{-9}$ fitted beforehand to amyloid aggregation scaling laws (Figure 2), and $R^{*}=5.7$ nm, $k_{+}\approx k_{a}$ and $k_{2}\approx 0$; lines from top to bottom $k_{a}=4.13\times 1.2$ h^−1^, $k_{a}=4.13\times 1.1$ h^−1^ and $k_{a}=4.13$ h^−1^. Solid line: solution of the discretized population balance taking into account the presence of pre-assembled clusters (Appendix B, Section B.2). (**E**) Measured (symbols) and simulated (lines) evolution of ${\bar{R}}_{h}$ during ataxin-3 aggregation (adapted from Silva et al. [17]). Lines: representations of Equation (4) using previously fitted values of $k_{a}$ and $k_{b}$, and $R^{*}=91$ nm, $R_{2}^{*}=15$ nm, $k_{2}\approx k_{a}$, $k_{+}\approx 0$ and (from top to bottom) $k_{a}/8$, $k_{a}/4$ and $k_{a}$ [17].

The previous models present a detailed picture of the different steps affecting the formation of the insoluble filaments that can be further tested using DLS measurements of particle size distributions (PSDs). Contrary to what was observed for ataxin-3 [17], the size of insulin fibrils tends to increase over time until reaching hydrodynamic radii ($R_{h}$) above the micrometer scale—Figure 4A–D (insulin) and Figure 4E (ataxin-3). This is not surprising taking into account the TEM images obtained at the end of each aggregation assay (Figure 1A,B), and the negligible role of the elongation step during ataxin-3 fibrillation. Another obvious difference to ataxin-3 is the persisting dominance of the left-side peak ($R_{h}<10$ nm) up to the end of the aggregation reaction (Figure 4A–C). To a certain extent, this is explained by the value of protein solubility ($C^{*}$), which as already discussed, is much higher in the case of human insulin. While the final concentration of soluble ataxin-3 was too low to be detected by DLS [17], the $C^{*}$ value of insulin is responsible for the population of soluble protein to continue predominating, even after large insoluble aggregates are formed (Figure 4C). Another reason explaining the modest increase in the intensity of scattered light of larger particles is associated with the dispersion of sizes and consequential broadening of PSDs provoked by the continuous elongation of old and newly-formed insulin fibrils, as opposed to the formation of ataxin-3 filaments with the constant dimension characteristic of the ataxin-3 secondary nucleus. In common with ataxin-3, fibril breakage has a minor role in determining the time variation of the PSD in quiescent insulin solutions: in the case of ataxin-3, the shape of these distributions did not change significantly during the burst and plateau phases of aggregation despite the increased relative importance of the population of ataxin-3 fibrils [17]. In the case of insulin, the elongation-dominated mechanism can be discerned from the expected fibril size increase during the burst phase (Figure 4A,B,D), whereas, after ~4.5 h incubation, the mean aggregate size stabilizes at a constant value of ${\bar{R}}_{h}\approx 1100$ nm without any visible signs of fibril fragmentation (Figure 4C,D).

The absence of significant fibril breakage reinforces the thesis that the oligomerization pathway is the main reason for the weak concentration dependence of the lag phase duration. Therefore, the alternative suggestion put forward by Knowles et al. [29] ascribing the less-than-linear scaling laws to predominant fibril breakage could not be confirmed in the cases of insulin and ataxin-3 aggregation. Owing to the negligible influx of new filaments created by fibril fragmentation, the discretized population balance adopted by the crystallization-like model (CLM) can be simplified to the following closed-form solution [17]:

(4) $$\;{\left(\frac{R^{*}}{{\bar{R}}_{h}}\right)}^{3}=\frac{k_{b}}{1-k_{b}}\left[\frac{\ln\left(1-\alpha \right)+k_{a}t}{\alpha \left(1-k_{b}\right)}\left(1-\frac{k_{2}}{k_{a}}{\left(\frac{R^{*}}{R_{2}^{*}}\right)}^{3}\right)-\left(1-\frac{k_{2}}{k_{a}k_{b}}{\left(\frac{R^{*}}{R_{2}^{*}}\right)}^{3}\right)\right]\;$$

with α given by Equation (1) and $R^{*}/R_{2}^{*}$ representing the ratio of hydrodynamic radii of primary and secondary nuclei. Interestingly, when the dominant autocatalytic process is fibril elongation ($k_{a}\approx k_{+}\gg k_{2}$), the value of $R^{*}$ can be estimated from the limiting case of Equation (4) for long reaction times (t→∞)

(5) $$\;{\left(\frac{R^{*}}{{\bar{R}}_{\infty }}\right)}^{3}=\frac{k_{b}}{1-k_{b}}\left(\frac{\ln\left(1/k_{b}\right)}{1-k_{b}}-1\right)\;$$

using the values of $k_{b}$ and final fibril size $({\bar{R}}_{\infty }$) as the only inputs. After replacing the values of $k_{b}=6.90\times 10^{-9}$ (fitted to the ThT aggregation data for $C_{T}=5$ mg/mL) and of ${\bar{R}}_{\infty }=1100$ nm (estimated by DLS) in Equation (5), the result of $R^{*}=5.7$ nm is obtained, which is a dimension slightly larger than the size of the insulin monomer. The contrast between this result and the critical size of 91 nm (corresponding to $\sim 1.5\times 10^{5}$ monomers) found for the initial ataxin-3 cluster (Figure 4E) indicates that the differences in the aggregation mechanism of the two proteins are already evident from the initial nucleation events. The higher entropic barrier that has to be overcome to generate the primary nucleus of ataxin-3 helps to explain why this phase transition process is so much slower than that of insulin.

Although our estimations are not sufficiently accurate to describe the exact aggregation state of the primary nucleus of insulin, it seems clear that only a few monomers are required to originate the fibrillar aggregates. Such predictions of the critical amyloid size can be affected by the existence of large contaminant particles interfering with the final size estimation used in Equation (5). In the present case, a well-defined distribution of particles possibly consisting of disordered protein clusters with $R_{h}$ between ~100 nm and >1000 nm is identified right from the beginning of the DLS measurements (Figure 4A,D). Next, we will show that its occurrence should not have affected the final PSDs. Differently from the emerging peak observed since the beginning of ataxin-3 aggregation [17], the initial size distributions shown for insulin in Figure 4A do not evolve in a clearly defined way until close to the burst phase of fibril elongation shown in Figure 4B. Pre-filtration of the insulin solution using 0.22 μm syringe filters efficiently removed these particles (Figure S2), but it also delayed the onset of the fibrillation process until a point where the kinetic measurements of Foderà et al. [26] could not be reproduced anymore. Therefore, pre-assembled protein clusters act as important heterogeneous nucleation centers without which the rapid formation of ordered aggregates is compromised [37,38,39]. The low concentration of the insulin clusters (fraction of total protein $<10^{-10}$ estimated from the initial PSDs) is high enough to conceal the initial progress of fibril sizes expected to start at $R^{*}=5.7$ nm and not from ${\bar{R}}_{h}$ values greater than 100 nm (compare dashed lines and experimental values in Figure 4D). In order to include the contribution of pre-existing assemblies in the predicted PSDs, numerical simulations were carried out as previously described for ataxin-3 [17], with the additional introduction of a simple mechanism of cluster-fibril adhesion described in detail in Section B.2 of Appendix B and in Figure S3. The challenge was to reproduce the experimental results in Figure 4A–D, namely, the initial presence of pre-assembled clusters, the gradual vanishing of this population as new insulin fibrils are formed, and the final emergence of a differentiated population of large aggregates. This was achieved using the values of $k_{a}$ and $k_{b}$ fitted to ThT aggregation data and one additional fitting parameter establishing the physical limit of particle detection (Figure 4D, solid line, and Figure S3A–C). The good agreement between theoretical and measured PSDs does not necessarily mean that cluster-fibril adhesion is the only mechanism capable of describing the size evolution of the initial clusters. In fact, since the numerical simulations assuming no pre-existing aggregates are still able to describe the later phase of fibril aggregation and the steady-state size distributions (Figure S3D–F), it is conceivable that the scarce population of clusters could have declined by means of other mechanisms, involving, for example, dissociation processes elicited by the decreasing concentration of dissolved protein. Although these hypotheses would imply the introduction of new model parameters such as cluster dissociation rate constants, the bottom line conclusion would remain that the final PSDs are negligibly affected by the presence of pre-assembled clusters.

To sum up, NMR, DLS and ThT aggregation data were used to conclude that soluble, partially oligomerized insulin gives rise to fibrillar aggregates by the processes of primary nucleation and subsequent fibril elongation with minor contributions from secondary nucleation and fibril breakage. A critical amyloid size of $R^{*}=5.7$ nm could be calculated for insulin using Equation (5) and the value of ${\bar{R}}_{\infty }$ estimated from the final PSD.

### 3.4. Systematization of Concepts

**Figure 5:**
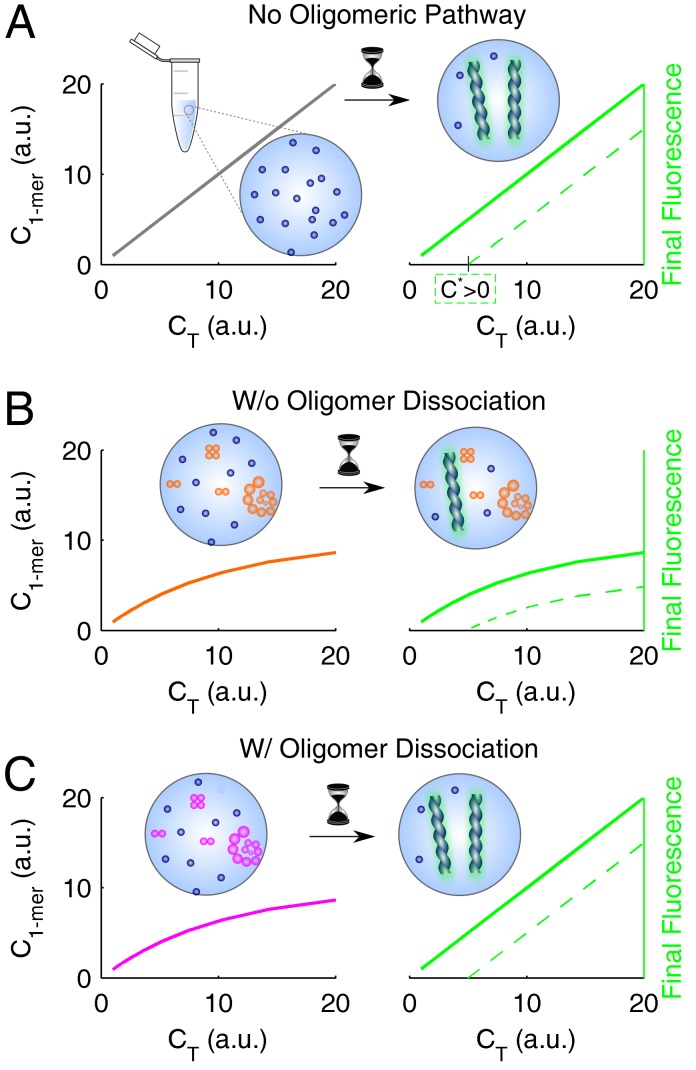
Equilibrium scaling laws used to unveil the oligomerization pathway. The initial distribution of monomer and total protein (left side) influences the end-point amyloid signal (right side); the correspondence is direct in the cases of (**A**) no oligomerization pathway and (**B**) irreversible oligomerization, and indirect in the case of (**C**) fully reversible oligomerization. Green lines represent cases of protein solubility values $C^{*}=0$ (solid lines) and $C^{*}>0$ (dashed lines).

**Figure 6:**
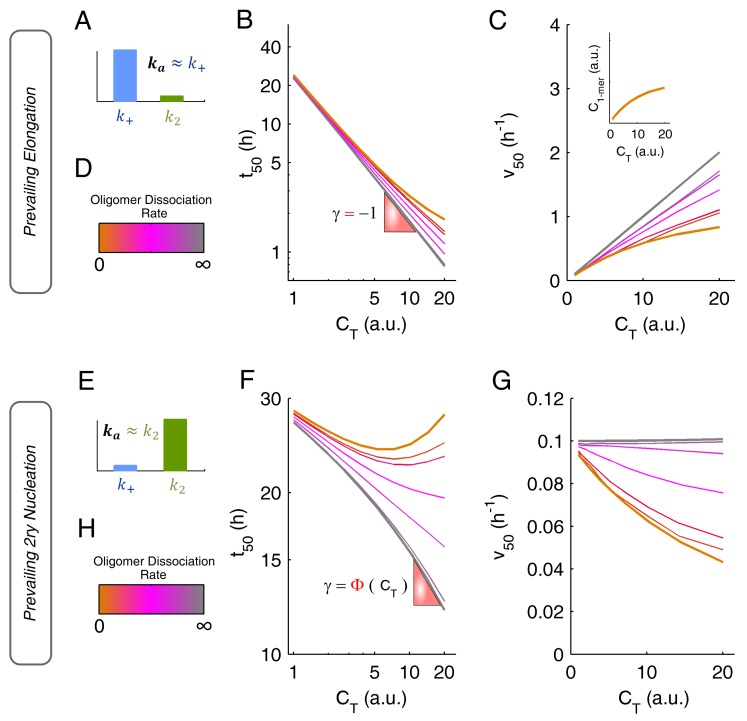
Impact of oligomerization on amyloid fibrillation kinetics when the dominant autocatalytic step is either fibril elongation (top) or secondary nucleation (bottom). (**A**–**D**) If fibril elongation is prevalent, then $k_{a}\approx k_{+}$ (**A**) and the scaling laws of $t_{50}$ (**B**, double-logarithmic plot) and $v_{50}$ (**C**) can change from linear to markedly nonlinear depending on the rate of oligomer dissociation (**D**). (**C**) Inset: in the case of irreversible oligomerization (orange lines), the scaling laws of $v_{50}$ reflect the (effective) initial concentration of monomeric protein. (**E**–**H**) If secondary nucleation is prevalent, then $k_{a}\approx k_{2}$ (**E**) and the concentration dependences of $t_{50}$ (**F**, double-logarithmic plot) and $v_{50}$ (**G**) are either poorly defined or markedly nonlinear according to the rate of oligomer dissociation (**H**).

The conclusions drawn for insulin and ataxin-3 are expected to generalize well, not only because they are supported by a combination of complementary results (obtained, in the case of insulin, by two other research teams besides our own), but also as a consequence of the wide spectrum of behaviors covered by the two systems: from nearly irreversible (insulin) to fully reversible (ataxin-3) oligomerization, and from dominant elongation (insulin) to dominant secondary nucleation (ataxin-3). A linkage between the occurrence of soluble oligomers and amyloid fibrillation kinetics can be established from the analysis of equilibrium and kinetic proportionality relations, as summarized in Figure 5 and Figure 6, respectively. The direct proportion of the end-point amyloid signal and protein concentration predicted in the absence of the oligomerization pathway (Figure 5A), will not be observed in the cases of refractory or slow dissociating oligomers (Figure 5B). If the formation of amyloid fibrils is capable of totally reversing the oligomerization equilibrium (Figure 5C), the $F_{F}$ signal would not differ substantially from that of fully dissociated protein.

The building evidence associating soluble oligomers to the pathogenesis of neurodegenerative diseases allows us to anticipate a new interest in chemical kinetic analysis as a tool to identify potential modulators of off-pathway oligomerization. In this respect, the final fluorescence value is a direct measurement of the extent of the amyloidogenic reaction but it can also reveal whether parallel aggregation pathways are inhibited or promoted by test compounds. For example, IAPP mimics synthesized with N-methylated amide bonds inhibit the aggregation of IAPP and Aβ40 by stabilizing protein monomers and nontoxic oligomers, thereby shifting the equilibrium towards the production of less amyloid fibrils and eliciting lower $F_{F}$ values [40]. Although complementary measurements are required in order to validate oligomer modulation effects, final fluorescence analysis is well suited for primary screenings of large libraries of chemical compounds.

The oligomerization pathway can be further probed by the analysis of kinetic scaling laws, which have different interpretations according to whether the autocatalytic step is fibril elongation (Figure 6A–D) or secondary nucleation (Figure 6E–H). In both cases, however, marked deviations from linearity are obtained in the presence of slowly dissociating oligomers. Absolute values of the scaling factor |γ| lower than 1 are admissible independently of the dominant secondary step (Figure 6B,F). If a high degree of oligomerization persists during amyloid fibril formation, positive $t_{50}$ vs. $C_{T}$ dependences are also possible, especially when secondary nucleation is a predominant step (Figure 6F). The aggregation rate $v_{50}$ is a useful comparator to gauge the kinetic impact of soluble oligomers based on marked deviations from the straight-line relationships (Figure 6C,G), but also to identify cases of dominant fibril elongation (positive $C_{T}$-dependence) and dominant secondary nucleation (neutral or negative $C_{T}$-dependence).

## 4. Conclusions

In conclusion, sigmoidal shapes of ThT fluorescence aggregation curves of insulin and ataxin-3 indicated that primary nucleation is the rate limiting step of amyloid fibril formation in both model proteins. Unconventionally weak $t_{50}$ scaling with protein concentration was explained by different aggregation mechanisms, involving, in one case (ataxin-3), dissociable soluble oligomers and rapid secondary nucleation, and in the other (insulin), refractory soluble oligomers and rapid fibril elongation. This was inferred from the analysis of the often disregarded measurables of end-point fluorescence and half-life aggregation rate, and could be confirmed by DLS and NMR results without overparameterization issues. Over and above the importance of reaction scaling laws to discriminate the mechanisms of protein aggregation, the rationale presented here is originally oriented to the discovery of new drugs targeting soluble oligomers. This compelling therapeutic target in neurodegenerative diseases [1,2,7], as well as in type 2 diabetes [41,42], remains largely unexplored except for very recent and encouraging candidate antibody therapies [43,44]. With the new chemical kinetic toolbox, amyloid binding assays can be utilized in either high-throughput screenings or drug repurposing strategies in the quest for disease-modifying, anti-oligomerization compounds.

## Supplementary Materials

The following are available online at http://www.mdpi.com/2218-273X/8/4/108/s1, Figure S1: Overfitting can be misleading even if good fitting statistics are obtained, Figure S2: Pre-assembled clusters present in fresh insulin solutions are removed upon filtration, Figure S3: Measured and simulated size distributions of insulin aggregates represented in normalized units of scattered light intensity.

## Appendix A. Model Equations

### Appendix A.1. Closed-Form Model Equations

Closed-form solutions of the CLM can be obtained whenever the phase transition process (in this case, amyloid fibril formation) is the only pathway by which the soluble protein is consumed [10,12,17,45]. The formation of insoluble species begins with a primary nucleation step (rate constant $k_{n}$) and then proceeds through the occurrence of the secondary nucleation and growth steps (rate constants $k_{2}$ and $k_{+}$, respectively). Fibril breakage (rate constant $k_{-}$) increases the total number of filaments but leaves their overall mass unchanged [17]. Thermodynamic equilibrium is attained when the chemical potential of the solution equals that of the insoluble fraction. At that point, the concentration of soluble protein corresponds to the protein solubility and, therefore, supersaturation $\Delta C=C-C^{*}$ (or in dimensionless form $\sigma =\Delta C/C^{*}$) is zero. Supersaturation, the thermodynamic driving force for phase transition, is alternatively expressed in terms of reaction conversion (α):

(A1) $$\;\sigma =\sigma_{0}\left(1-\alpha \right)\;$$

The differential form of the CLM equation [12]

(A2) $$\;\frac{d\alpha }{dt}=k_{n}{\left(1-\alpha \right)}^{2}+\left(k_{+}+k_{2}\right)\left(1-\alpha \right)\alpha \;$$

has the following analytical solution for the case of unseeded reactions ($\alpha_{0}=0$):

(A3) $$\;\alpha =1-\frac{1}{k_{b}\left[\exp\left(k_{a}t\right)-1\right]+1}\;$$

where $k_{a}=k_{+}+k_{2}$ and $k_{b}=k_{n}/k_{a}$. The half-life coordinates $t_{50}$ and $v_{50}$ are obtained by setting α=0.5 in Equations (A2) and (A3), respectively:

(A4a) $$\;t_{50}=\frac{1}{k_{a}}\ln\left(\frac{1}{k_{b}}+1\right)\;$$

(A4b) $$\;v_{50}=\frac{k_{a}}{4}\left(k_{b}+1\right)\;$$

### Appendix A.2. Oligomerization Equilibrium of Insulin

For insulin aggregation, the formation and dissociation of soluble oligomers is assumed to take place in a different timescale than that of amyloid fibril formation. Although soluble insulin is distributed over different oligomeric states, only the concentration of monomers is directly depleted by the formation of amyloid fibrils. The progress of amyloid signal with time is still described by Equation (A3), while the analysis of kinetic scaling laws requires supersaturation to be expressed in terms of monomer concentration ($C_{1}$). To estimate the relationship between the initial value of $C_{1}$ and the total insulin concentration ($C_{T}$), the isodesmic-type oligomerization equilibrium of insulin determined by Bocian et al. [21] (Figure 2B) was followed:

(A5) $$\;C_{T}=C_{1}+2K_{12}C_{1}^{2}+4K_{12}^{2}K_{24}C_{1}^{4}+\overset{\infty }{\underset{n=3}{\sum }}2nK_{24}K_{46}K_{iso}^{n-3}K_{12}^{n}C_{1}^{2n}.\;$$

### Appendix A.3. Oligomerization Equilibrium of Ataxin-3

The oligomerization reaction scheme of ataxin-3 involves successive reversible steps of monomer addition (Figure 2A), each n-step being characterized by the rate constants of monomer aggregation ($\kappa_{n+}$) and dissociation ($\kappa_{n-}$). Except for the initial dimerization reaction (rate constants $\kappa_{1+}$ and $\kappa_{1-}$), all the subsequent steps are well characterized by the same fixed values of $\kappa_{n+}$ and $\kappa_{n-}$. Since the soluble oligomers do not participate in the formation of amyloid fibrils, the concentration of the n-mer ($C_{n}$) is solely determined by the linear equilibrium balance, whereas the concentration of monomer is also dictated by the rate of amyloid fibrillation (dM/dt):

(A6) $$\begin{array}{c} \;\frac{dC_{1}}{dt}=-\frac{dM}{dt}-\kappa_{1+}C_{1}^{2}+\kappa_{1-}C_{2}+\overset{\infty }{\underset{i=2}{\sum }}\left(-\kappa_{n+}C_{1}C_{i}+\kappa_{n-}C_{i+1}\right)\;\\ \;\frac{dC_{2}}{dt}=\kappa_{1+}C_{1}^{2}-\kappa_{1-}C_{2}-\kappa_{n+}C_{1}C_{2}+\kappa_{n-}C_{3}\;\\ \;\frac{dC_{3}}{dt}=\kappa_{n+}C_{1}C_{2}-\kappa_{n-}C_{3}-\kappa_{n+}C_{1}C_{3}+\kappa_{n-}C_{4}\;\\ \vdots \\ \;\frac{dC_{n}}{dt}=\kappa_{n+}C_{1}C_{i-1}-\kappa_{n-}C_{n}-\kappa_{n+}C_{1}C_{n}+\kappa_{n-}C_{n+1}\; \end{array}$$

with the sum term accounting for the capture and release of one protein molecule per elementary step. After expressing supersaturation in terms of monomer concentration, the CLM Equation (A2) is reformulated as [17]:

(A7) $$\;\frac{dM}{dt}=k_{a}k_{b}{\left(\frac{C_{1}-C^{*}}{C_{T}-C^{*}}\right)}^{2}M_{F}+k_{a}\left(\frac{C_{1}-C^{*}}{C_{T}-C^{*}}\right)M\;$$

where $M_{F}=C_{T}-C^{*}$ corresponds to the final concentration of monomers present in the insoluble phase if all soluble $n_{\mathrm{mers}}$ become dissociated during the process of amyloid fibrillation.

## Appendix B. Numerical Methods

### Appendix B.1. Scaling Laws

The kinetic scaling laws of insulin fibrillation were analyzed as follows: Equation (A5) was numerically solved for different values of total insulin concentration ($C_{T})$ using previously determined equilibrium constants ($K_{12}=4.9\times 10^{5}$, $K_{24}=5.0\times 10^{4}$, $K_{46}=2.7\times 10^{3}$ and $K_{\mathrm{iso}}=1.35\times 10^{4}$) [21]. The obtained values of monomer concentration (Figure 2C) were used to estimate the amyloid solubility of insulin ($C^{*}=0.029$ mg/mL) from the scaling law of the end-point ThT fluorescence with $C_{T}$ (Figure 2D). After recognizing fibril elongation as the predominant autocatalytic step during insulin fibrillation ($k_{a}\approx k_{+}$ and $k_{b}\approx k_{n}/k_{+}$), the dependence of the two CLM parameters on supersaturation was expressed as $k_{a}=k_{a}^{\prime }\Delta C$ and $k_{b}=k_{b}^{\prime }\Delta C$, with $\Delta C=C_{1}-C^{*}$. These definitions were replaced in Equations (A4a) and (A4b), which were then solved using the measured values of $t_{50}$ and $v_{50}$ (Figure 2E,F, respectively). Finally, the values of $k_{a}^{\prime }$ and $k_{b}^{\prime }$ estimated for each insulin concentration were averaged to obtain $k_{a}^{\prime }=1.34\times 10^{2}$ mL/mg/h and $k_{b}^{\prime }=2.41\times 10^{-7}$ mL/mg.

In the case of ataxin-3, the set of differential equations comprising Equations (A6) and (A7) were numerically solved using Mathworks^®^ MATLAB 2016b (Natick, MA, USA) to obtain the concentration of polymerized monomers (M) as a function of time for the cases of $C_{T}=10$ μM, 7 μM, 5 μM, 4 μM and 2 μM. In order to keep Equation (A6) manageable for numerical computation, a cut-off size of $n_{\infty }=9\times 10^{6}$ monomers was adopted as the maximum dimension of soluble ataxin-3 oligomers, and the condition $dC_{n}/dt=0$ was imposed for $n\geq n_{\infty }$. The initial conditions were set assuming no fibrillar aggregates present in solution (M(0)=0) and that the fractional compositions of monomers and n-mers correspond to those extracted from DLS measurements [17]. Since the predominant autocatalytic step during ataxin-3 fibrillation is secondary nucleation, $k_{a}\approx k_{2}$ and $k_{b}\approx k_{n}/k_{2}$. Owing to the low solubility of ataxin-3 ($C^{*}\approx 0$), the initial supersaturation is a direct proportion of $C_{T}$ and the CLM parameters are given as $k_{a}=k_{a}^{\prime }C_{T}^{n_{2}}$ and $k_{b}=k_{b}^{\prime }C_{T}^{2-n_{2}}$. The proportionality constants $k_{2}^{\prime }$ and $k_{n}^{\prime }$, and the order-of-reaction $n_{2}$ were estimated by minimizing the absolute error between predicted and measured ThT fluorescence (F) progress curves using the experimentally determined calibration curve F(a.u.)=0.70×M(μM) and the known oligomerization rate constants $\kappa_{1+}=7.99\times 10^{-4}$ μM^−1^h^−1^, $\kappa_{1-}=9.73$ h^−1^, $\kappa_{n+}=0.167$ μM^−1^h^−1^, and $\kappa_{n-}=0.775$ h^−1^ [17].

To simulate the $t_{50}$ and $v_{50}$ scaling laws in Figure 6, Equations (A6) and (A7) were numerically solved as described for ataxin-3 using illustrative values of $C^{*}=0$, $k_{a}=0.4C_{T}^{n_{2}}$ h^−1^ and $k_{b}=5\times 10^{-4}{\left(C_{T}/5\right)}^{2-n_{2}}$. Two limit situations corresponding to predominant elongation step ($n_{2}=1$) or predominant secondary nucleation ($n_{2}=0$) processes were considered. Soluble protein was admitted to occur either as a monomer or as a dimer ($\kappa_{n+}=0$ and $\kappa_{n-}=0$) with the distribution of initial species given by the exemplar function $C_{1}=10\times \left(1-e^{-C_{T}/10}\right)$ for $C_{T}$ values comprised between 0 and 20 in arbitrary concentration units. The relative weight of the oligomer dissociation rate was investigated by changing the value of $\kappa_{1-}$ between 0 and 5 h^−1^ while keeping a fixed value of $\kappa_{1+}=0$. The normalized amyloid signal ($M/M_{F}$) was computed over time and the corresponding half-life coordinates, $t_{50}$ and $v_{50}$ were represented as a function of $C_{T}$ (Figure 6).

### Appendix B.2. Discretized Population Balance

The time evolution of the size distribution of insulin fibrils was simulated using the discrete population balance derived for general phase transition processes comprising the steps of primary nucleation, secondary nucleation, growth/elongation and breakage [12,17]. As previously described for ataxin-3, the concentration of filaments composed by j monomers ($f_{j}$) is given as [17]:

(A8) $$\begin{array}{c} \;\frac{df_{j}}{dt}=\frac{1}{j}\left[k_{n}{\left(1-\alpha \right)}^{2}M_{\infty }\delta_{j,n^{*}}+k_{2}\left(1-\alpha \right)\alpha M_{\infty }\delta_{j,n_{2}^{*}}\right]+\;\\ \;+k_{+}\left(1-\alpha \right)\left(j-1\right)f_{j-1}-k_{+}\left(1-\alpha \right)jf_{j}+\;\\ \;+\frac{1}{j}\left[-k_{-}f_{j}\left(j-n_{2}^{*}\right)ℋ\left(j-2n^{*}-1\;\right)+\overset{\infty }{\underset{i=j+1}{\sum }}k_{-}f_{i}ℋ\left(i-2n^{*}-1\right)\right]\; \end{array}$$

The Kronecker delta functions in Equation (A8) set the sizes of the primary and secondary nuclei to fixed values of $n^{*}$ and $n_{2}^{*}$, respectively, with the latter being adopted as the smallest possible filament size ($j\geq n_{2}^{*}$). The Heaviside function establishes a minimum fibril size of $2n^{*}+1$ molecules above which fragmentation starts to occur [46]. In the case of insulin, this equation is simplified since secondary nucleation and fibril breakage take place to a negligible extent ($k_{2}\approx 0$ and $k_{-}\approx 0$). The previous Equation (A2) can be obtained from Equation (A8) by extending the sum of $j\times df_{j}/dt$ to all filaments [17]:

(A9) $$\;\frac{dM}{dt}=\overset{\infty }{\underset{j=n_{2}^{*}}{\sum }}j\frac{df_{j}}{dt}\;$$

A population of pre-existing clusters with hydrodynamic radii $R_{h}$ >100 nm was identified in the analyzed insulin solutions (Figure S2). The DLS intensity-peak corresponding to this population gradually vanished from the measured size distributions as fibril elongation took place (Figure 4A–D). To simulate this behavior, a simple particle adhesion mechanism is proposed in which pre-existing clusters composed by k monomers are considered to join to the available j-mer fibrils and form heterogeneous agglomerates composed by l=k+j monomers. During the initial phases of the reaction, a number of pre-existing clusters remains isolated because there are fewer fibrils than clusters. This scenario is then reversed as newly formed fibrils outnumber the initial clusters. Therefore, the less abundant species are the ones dictating the number of aggregates that will participate in the adhesion process ($P_{a}$):

(A10) $$\;P_{a}=\min(P=\overset{\infty }{\underset{j}{\sum }}f_{j},P_{c}\left(0\right)=\overset{\infty }{\underset{k}{\sum }}f_{k}\left(0\right))\;$$

where P is the concentration of insulin fibrils, $P_{c}\left(0\right)\approx 5.5\times 10^{-16}$ M is the total concentration of clusters and $f_{k}\left(0\right)$ is the concentration of k-mer clusters—$P_{c}\left(0\right)$ and $f_{k}\left(0\right)$ are estimated from the initial DLS measurements. At a given instant, the number of k-mer clusters joining to j-mer fibrils is a function of the relative amounts of each type of aggregates,

(A11) $$\;f_{k\to j}=P_{a}\times \left[\frac{f_{j}}{P}\right]\times \left[\frac{f_{k}\left(0\right)}{P_{c}\left(0\right)}\right],\;$$

meaning that the concentrations of isolated fibrils ($f_{j}^{0}$) and isolated clusters ($f_{k}^{0}$) are

(A12) $$\;f_{j}^{0}=f_{j}-\overset{\infty }{\underset{k}{\sum }}f_{k\to j},\;$$

(A13) $$\;f_{k}^{0}=f_{k}\left(0\right)-\overset{\infty }{\underset{j}{\sum }}f_{k\to j},\;$$

and that the concentration of heterogeneous agglomerates composed by a total l monomers ($f_{l}^{1}$) is

(A14) $$\;f_{l}^{1}=\overset{\infty }{\underset{k}{\sum }}\overset{\infty }{\underset{j}{\sum }}f_{k\to j}\delta_{k+j,l}.\;$$

Here, the Kronecker delta function is used to limit the sum terms to the possible adhesion contacts producing l-mer agglomerates. Finally, the concentration of all l-mer aggregates (${\bar{f}}_{l}$) is given as:

(A15) $$\;\bar{f_{l}}=f_{j=l}^{0}+f_{k=l}^{0}+f_{l}^{1},\;$$

which can be used to compute the theoretical size distributions. It follows from the proposed one-to-one adhesion mechanism that

(A16) $$\;P_{a}=\overset{\infty }{\underset{l}{\sum }}f_{l}^{1},\;$$

and that the total number of scattering particles corresponds to the concentration of the most abundant species:

(A17) $$\;\overset{\infty }{\underset{l}{\sum }}\bar{f_{l}}=\max\left(P,P_{c}\left(0\right)\right).\;$$

The following additional approximations were adopted during the derivation of this model: (i) fibril elongation is unaffected by the presence of joined clusters, (ii) complex adhesion pathways are not considered and (iii) the presence of isolated fibrils and isolated clusters is a necessary and sufficient condition for productive adhesion. Other mechanisms explaining the observed decline of the cluster population are admissible, e.g., by admitting progressive cluster dissociation. The goal of describing the experimental results in Figure 4A–D could, however, be achieved using previously determined values of $k_{a}$ and $k_{b}$ (Figure S3A–C) and adopting the physical limit of particle detection ($\bar{f_{l}}>1.6\times 10^{-23}$ M) as the only adjustable parameter set to minimize the differences between the measured DLS data and the theoretical size distributions. Mathworks^®^ MATLAB R2013b was used to numerically solved Equation (A8) and Equations (A10)–(A15) for a cut-off size of isolated insulin fibrils of $n=1\times 10^{13}$ monomers. The obtained $\bar{f_{l}}\left(t\right)$ profiles were converted into intensity-based size distributions following the Rayleigh law of light scattering [17]. Importantly, the final size distributions were not significantly affected by the presence of initial clusters as indicated by the simulated results for $P_{c}\left(0\right)=0$ (Figure S3D–F).

## References

[1] David B., Hayer-Hartl M., Hartl F.U. (2016). In Vivo Aspects of Protein Folding and Quality Control. Science.

[2] Selkoe D.J., Hardy J. (2016). The Amyloid Hypothesis of Alzheimer’s Disease at 25 years. EMBO Mol. Med..

[3] Wienkers L.C., Heath T.G. (2005). Predicting In Vivo Drug Interactions from In Vitro Drug Discovery Data. Nat. Rev. Drug Discov..

[4] Kundel F., Tosatto L., Whiten D.R., Wirthensohn D.C., Horrocks M.H., Klenerman D. (2018). Shedding Light on Aberrant Interactions: A Review of Modern Tools for Studying Protein Aggregates. FEBS J..

[5] Breydo L., Uversky V.N. (2015). Uversky. Structural, Morphological, and Functional Diversity of Amyloid Oligomers. FEBS Lett..

[6] Young L.M., Ashcroft A.E., Radford S.E. (2017). Small Molecule Probes of Protein Aggregation. Curr. Opin. Chem. Biol..

[7] Benilova I., Karran E., De Strooper B. (2012). The Toxic Aβ Oligomer and Alzheimer’s Disease: An Emperor in Need of Clothes. Nat. Neurosci..

[8] Lee S.J., Nam E., Lee H.J., Savelieff M.G., Lim M.H. (2017). Towards an Understanding of Amyloid-β Oligomers: Characterization, Toxicity Mechanisms, and Inhibitors. Chem. Soc. Rev..

[9] Arosio P., Vendruscolo M., Dobson C.M., Knowles T.P. (2014). Chemical Kinetics for Drug Discovery to Combat Protein Aggregation Diseases. Trends Pharmacol. Sci..

[10] Crespo R., Villar-Alvarez E., Taboada P., Rocha F.A., Damas A.M., Martins P.M. (2016). What Can the Kinetics of Amyloid Fibril Formation Tell about Off-Pathway Aggregation?. J. Biol. Chem..

[11] Padrick S.B., Miranker A.D. (2002). Islet Amyloid: Phase Partitioning and Secondary Nucleation Are Central to the Mechanism of Fibrillogenesis. Biochemistry.

[12] Crespo R., Rocha F.A., Damas A.M., Martins P.M. (2012). A Generic Crystallization-Like Model That Describes the Kinetics of Amyloid Fibril Formation. J. Biol. Chem..

[13] Meisl G., Yang X., Hellstrand E., Frohm B., Kirkegaard J.B., Cohen S.I., Dobson C.M., Linse S., Knowles T.P. (2014). Differences in Nucleation Behavior Underlie the Contrasting Aggregation Kinetics of the Aβ40 and Aβ42 Peptides. Proc. Natl. Acad. Sci. USA.

[14] Meisl G., Kirkegaard J.B., Arosio P., Michaels T.C., Vendruscolo M., Dobson C.M., Linse S., Knowles T.P. (2016). Molecular Mechanisms of Protein Aggregation from Global Fitting of Kinetic Models. Nat. Protoc..

[15] Eden K., Morris R., Gillam J., MacPhee C.E., Allen R.J. (2015). Competition between Primary Nucleation and Autocatalysis in Amyloid Fibril Self-Assembly. Biophys. J..

[16] Morris R.J., Eden K., Yarwood R., Jourdain L., Allen R.J., MacPhee C.E. (2013). Mechanistic and Environmental Control of the Prevalence and Lifetime of Amyloid Oligomers. Nat. Commun..

[17] Silva A., Almeida B., Fraga J.S., Taboada P., Martins P.M., Macedo-Ribeiro S. (2017). Distribution of Amyloid-Like and Oligomeric Species from Protein Aggregation Kinetics. Angew. Chem. Int. Ed..

[18] Finkelstein A.V., Dovidchenko N.V., Galzitskaya O.V. (2018). What is Responsible for Atypical Dependence of the Rate of Amyloid Formation on Protein Concentration: Fibril-Catalyzed Initiation of New Fibrils or Competition with Oligomers?. J. Phys. Chem. Lett..

[19] Banerjee S., Sun Z., Hayden E.Y., Teplow D.B., Lyubchenko Y.L. (2017). Nanoscale Dynamics of Amyloid β-42 Oligomers as Revealed by High-Speed Atomic Force Microscopy. ACS Nano.

[20] Nielsen L., Frokjaer S., Brange J., Uversky V.N., Fink A.L. (2001). Probing the Mechanism of Insulin Fibril Formation with Insulin Mutants. Biochemistry.

[21] Bocian W., Sitkowski J., Tarnowska A., Bednarek E., Kawȩcki R., Koźmiński W., Kozerski L. (2008). Direct Insight into Insulin Aggregation by 2D NMR Complemented by PFGSE NMR. Proteins Struct. Funct. Bioinf..

[22] Vestergaard B., Groenning M., Roessle M., Kastrup J.S., Van De Weert M., Flink J.M., Frokjaer S., Gajhede M., Svergun D.I. (2007). A Helical Structural Nucleus is the Primary Elongating Unit of Insulin Amyloid Fibrils. PLoS Biol..

[23] Scarff C.A., Almeida B., Fraga J., Macedo-Ribeiro S., Radford S.E., Ashcroft A.E. (2015). Examination of Ataxin-3 (Atx-3) Aggregation by Structural Mass Spectrometry Techniques: A Rationale for Expedited Aggregation Upon Polyglutamine (polyQ) Expansion. Mol. Cell. Proteom..

[24] Ellisdon A.M., Thomas B., Bottomley S.P. (2006). The Two-Stage Pathway of Ataxin-3 Fibrillogenesis Involves a Polyglutamine-Independent Step. J. Biol. Chem..

[25] Provencher S.W. (1982). A Constrained Regularization Method for Inverting Data Represented by Linear Algebraic or Integral Equations. Comput. Phys. Comm..

[26] Fodera V., Librizzi F., Groenning M., Van De Weert M., Leone M. (2008). Secondary Nucleation and Accessible Surface in Insulin Amyloid Fibril Formation. J. Phys. Chem. B.

[27] Serrano A.L., Lomont J.P., Tu L.H., Raleigh D.P., Zanni M.T. (2017). A Free Energy Barrier Caused by the Refolding of an Oligomeric Intermediate Controls the Lag Time of Amyloid Formation by hIAPP. J. Am. Chem. Soc..

[28] Jayaraman M., Kodali R., Sahoo B., Thakur A.K., Mayasundari A., Mishra R., Peterson C.B., Wetzel R. (2012). Slow Amyloid Nucleation Via α-Helix-Rich Oligomeric Intermediates in Short Polyglutamine-Containing Huntingtin Fragments. J. Mol. Biol..

[29] Knowles T.P., Waudby C.A., Devlin G.L., Cohen S.I., Aguzzi A., Vendruscolo M., Terentjev E.M., Welland M.E., Dobson C.M. (2009). An Analytical Solution to the Kinetics of Breakable Filament Assembly. Science.

[30] Bernacki J.P., Murphy R.M. (2009). Model Discrimination and Mechanistic Interpretation of Kinetic Data in Protein Aggregation Studies. Biophys. J..

[31] Ditlev J.A., Mayer B.J., Loew L.M. (2013). There Is More Than One Way to Model an Elephant. Experiment-Driven Modeling of the Actin Cytoskeleton. Biophys. J..

[32] Wang G., Fersht A.R. (2015). Mechanism of Initiation of Aggregation of P53 Revealed by Φ-Value Analysis. Proc. Natl. Acad. Sci. USA.

[33] Lindberg D.J., Wenger A., Sundin E., Wesén E., Westerlund F., Esbjörner E.K. (2017). Binding of Thioflavin-T to Amyloid Fibrils Leads to Fluorescence Self-Quenching and Fibril Compaction. Biochemistry.

[34] Xue C., Lin T.Y., Chang D., Guo Z. (2017). Thioflavin-T as an Amyloid Dye: Fibril Quantification, Optimal Concentration and Effect on Aggregation. R. Soc. Open Sci..

[35] Jeong J.S., Ansaloni A., Mezzenga R., Lashuel H.A., Dietler G. (2013). Novel Mechanistic Insight into the Molecular Basis of Amyloid Polymorphism and Secondary Nucleation During Amyloid Formation. J. Mol. Biol..

[36] Agarwal V., Peters B. (2014). Solute Precipitate Nucleation: A Review of Theory and Simulation Advances. Advances in Chemical Physics: Volume 155.

[37] Lomakin A., Chung D.S., Benedek G.B., Kirschner D.A., Teplow D.B. (1996). On the Nucleation and Growth of Amyloid Beta-Protein Fibrils: Detection of Nuclei and Quantitation of Rate Constants. Proc. Natl. Acad. Sci. USA.

[38] Parmar A.S., Gottschall P.E., Muschol M. (2007). Pre-Assembled Clusters Distort Crystal Nucleation Kinetics in Supersaturated Lysozyme Solutions. Biophys. Chem..

[39] Ferreira C., Barbosa S., Taboada P., Rocha F.A., Damas A.M., Martins P.M. (2017). The Nucleation of Protein Crystals as a Race against Time with On- and Off-Pathways. J. Appl. Cryst..

[40] Yan L.M., Velkova A., Tatarek-Nossol M., Rammes G., Sibaev A., Andreetto E., Kracklauer M., Bakou M., Malideli E., Göke B. (2013). Selectively N-Methylated Soluble IAPP Mimics as Potent IAPP Receptor Agonists and Nanomolar Inhibitors of Cytotoxic Self-Assembly of Both IAPP and Aβ40. Angew. Chem. Int. Ed..

[41] Birol M., Kumar S., Rhoades E., Miranker A.D. (2018). Conformational Switching within Dynamic Oligomers Underpins Toxic Gain-of-Function by Diabetes-Associated Amyloid. Nat. Commun..

[42] Ke P.C., Sani M.A., Ding F., Kakinen A., Javed I., Separovic F., Davis T.P., Mezzenga R. (2017). Implications of Peptide Assemblies in Amyloid Diseases. Chem. Soc. Rev..

[43] Sevigny J., Chiao P., Bussière T., Weinreb P.H., Williams L., Maier M., Dunstan R., Salloway S., Chen T., Ling Y. (2016). The Antibody Aducanumab Reduces Aβ Plaques in Alzheimer’s Disease. Nature.

[44] Schenk D.B., Koller M., Ness D.K., Griffith S.G., Grundman M., Zago W., Soto J., Atiee G., Ostrowitzki S., Kinney G.G. (2017). First-in-Human Assessment of PRX002, an Anti—α-Synuclein Monoclonal Antibody, in Healthy Volunteers. Mov. Disord..

[45] Martins P.M. (2013). True and Apparent Inhibition of Amyloid Fibril Formation. Prion.

[46] Collins S.R., Douglass A., Vale R.D., Weissman J.S. (2004). Mechanism of Prion Propagation: Amyloid Growth Occurs by Monomer Addition. PLoS Biol..

