# Supplementary material for: Probing the Occurrence of Soluble Oligomers through Amyloid Aggregation Scaling Laws
Source: Biomolecules. 2018 Oct 4;8(4):108. doi: 10.3390/biom8040108 (PMC6316134; doi:10.3390/biom8040108)
Supplement: Supplementary file 1 [file biomolecules-08-00108-s001.pdf]

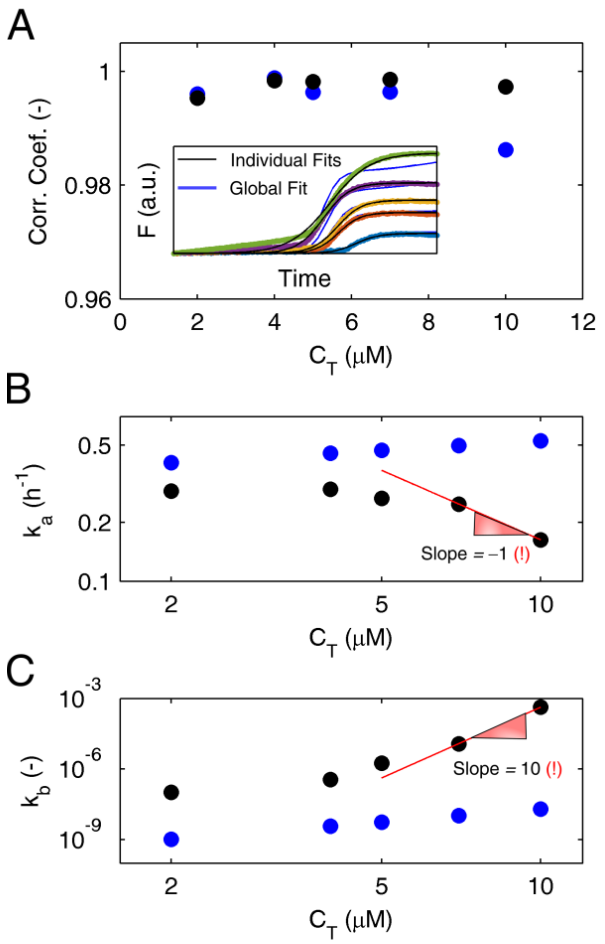

Figure S1. Overfitting can be misleading even if good fitting statistics are obtained. Comparison between individual, "oligomer-blind" fits (black symbols) and the global fit (blue symbols) described in Figure 3B. (A) Better correlation coefficients are, in general, obtained when the progress curves measured at different protein concentrations are analyzed individually. Inset: Experimental data (symbols) and numerical fits (lines) as described in Figure 3B. (B and C) Yet, when the oligomeric equilibrium is ignored, odd scaling laws are obtained in the double-logarithm representations of (B) k_a and (C) k_b as a function of total ataxin-3 concentration (C_T) (black symbols). Theories that miss the non-amyloidogenic pathways would require additional model parameters to explain (B) the negative k_a vs. C_T dependency or (C) the very high scaling coefficients of k_b (red lines). On the contrary, since the global fit takes into account the measured dissociation of ataxin-3 oligomers, good correlation coefficients could be obtained in (A) without having to elaborate on the physical model any further.

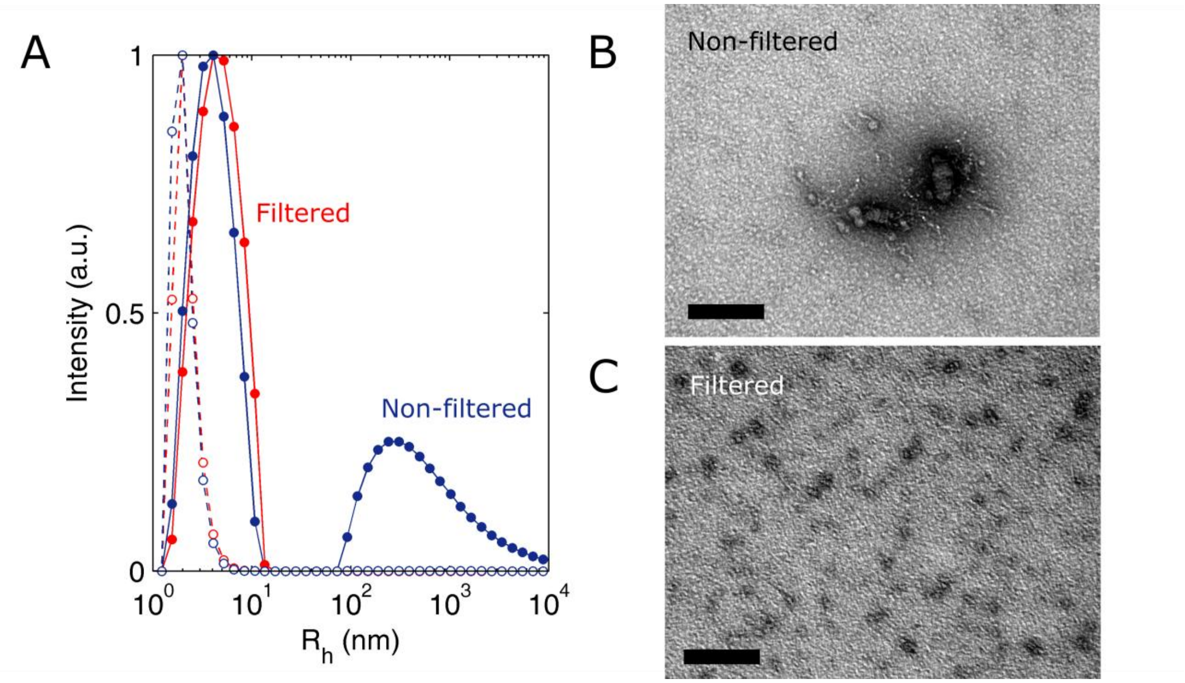

Figure S2. Pre-assembled clusters present in fresh insulin solutions are removed upon filtration. (A) Intensity-based (full symbols connected by solid lines) and number-based (open symbols connected by dashed lines) particle size distributions measured by DLS using non-filtered (blue) and filtered (red) insulin solutions. (B and C) Transmission electron microscopy micrographs of negatively stained aggregates present in non-filtered (B) and filtered (C) insulin solutions (scale bars, 200 nm).

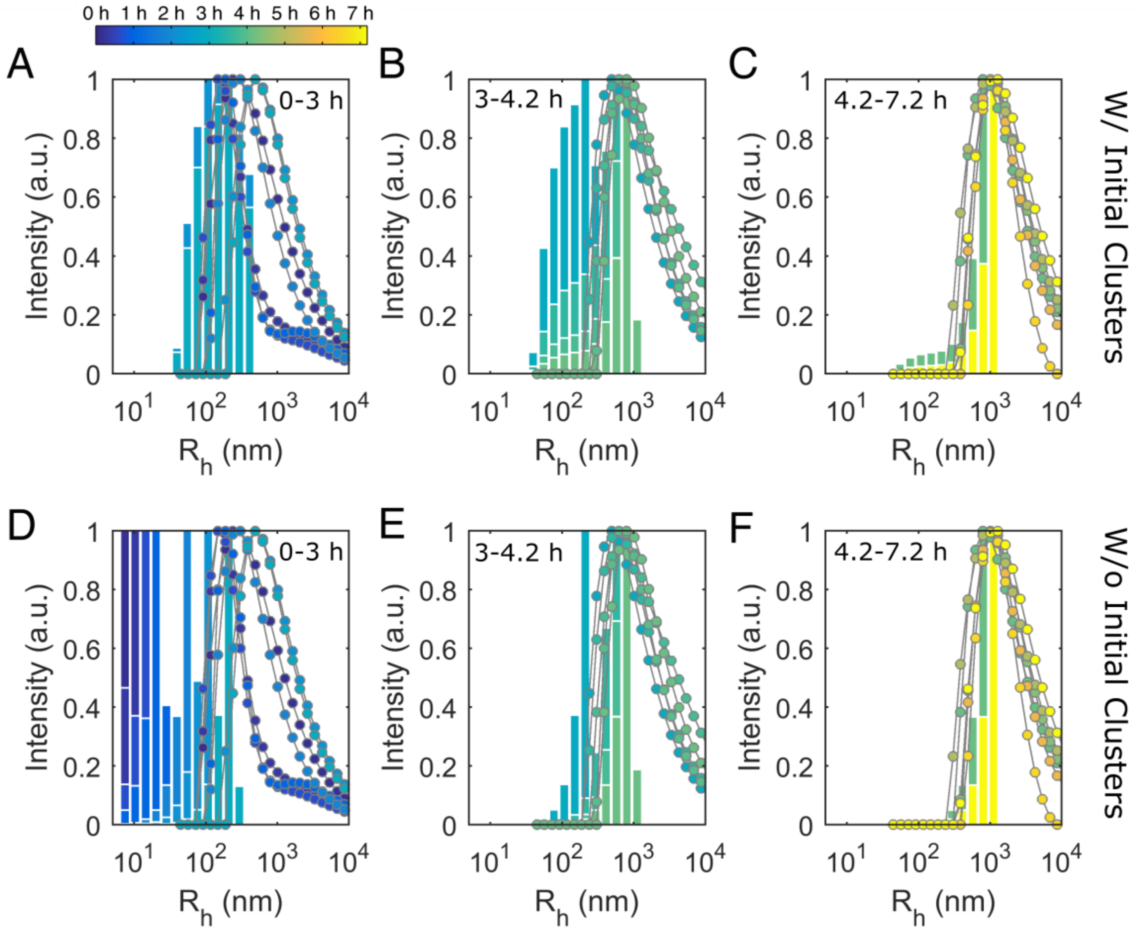

Figure S3. Measured (symbols connected by lines) and simulated (bars) size distributions of insulin aggregates represented in normalized units of scattered light intensity. Colors indicate different time points according to the color bar in (A). (A to C) The simulated results take into account the presence of pre-existing clusters and the subsequent one-to-one adhesion mechanism described in section S5 of Supplementary Text. (D to F) The occurrence of initial clusters was not considered during the numerical simulations.
